# Policy Rogue or Policy Entrepreneur? The Forms and Impacts of “Joined-Up Governance” for Child Health

**DOI:** 10.3390/children8030221

**Published:** 2021-03-13

**Authors:** Celine Cressman, Fiona A. Miller, Astrid Guttmann, John Cairney, Robin Z. Hayeems

**Affiliations:** 1 Better Outcomes Registry and Network (BORN Ontario), Children’s Hospital of Eastern Ontario Research Institute, Ottawa, ON K1H 8L1, Canada; celine.cressman@sickkids.ca; 2 Child Health Evaluative Sciences, Hospital for Sick Children, Toronto, ON M5G 1X8, Canada; astrid.guttmann@ices.on.ca (A.G.); robin.hayeems@sickkids.ca (R.Z.H.); 3 Institute of Health Policy, Management and Evaluation, University of Toronto, Toronto, ON M5T 3M6, Canada; 4 Institute for Clinical Evaluative Sciences (ICES), Sunnybrook Health Sciences Centre, Toronto, ON M4N 3M5, Canada; 5 Department of Paediatrics, Hospital for Sick Children, University of Toronto, Toronto, ON M5G 1X8, Canada; 6 School of Human Movement and Nutrition Sciences, University of Queensland, St Lucia, QLD 4072, Australia; j.cairney@uq.edu.au

**Keywords:** child, health policy, evidence, governance, health systems

## Abstract

Joined-up governance (JUG) approaches have gained attention as mechanisms for tackling wicked policy problems, particularly in intersectoral areas such as child health, where multiple ministries that deliver health and social services must collaborate if they are to be effective. Growing attention to the need to invest in early childhood to improve health and developmental trajectories, including through developmental screening, illustrate the challenges of JUG for child health. Using a comparative case study design comprised of the qualitative analysis of documents and key informant interviews, this work sought to explain how and why visible differences in policy choices have been made across two Canadian jurisdictions (Ontario and Manitoba). Specifically, we sought to understand two dimensions of governance (structure and process) alongside an illustrative example—the case of developmental screening, including how insiders viewed the impacts of governance arrangements in this instance. The two jurisdictions shared a commitment to evidence-based policy making and a similar vision of JUG for child health. Despite this, we found divergence in both governance arrangements and outcomes for developmental screening. In Manitoba, collaboration was prioritized, interests were aligned in a structured decision-making process, evidence and evaluation capacity were inherent to agenda setting, and implementation was considered up front. In Ontario, interests were not aligned and instead decision making operated in an opaque and siloed manner, with little consideration of implementation issues. In these contexts, Ontario pursued developmental screening, whereas Manitoba did not. While both jurisdictions aimed at JUG, only Manitoba developed a coordinated JUG system, whereas Ontario operated as a non-system. As a result, Manitoba’s governance system had the capacity to stop ‘rogue’ action, prioritizing investments in accordance with authorized evidence. In contrast, in the absence of a formal system in Ontario, policy ‘entrepreneurs’ were able to seize a window of opportunity to invest in child health.

## 1. Introduction

Preventive child health care is understood as a wicked policy problem because it involves complex social policy absent of definitive solutions [1,2]. Part of the complexity lies in the fact that child health straddles the boundaries of health and social policy and is not neatly situated within a single sector. Policy effort to enhance early child development (ECD), including reducing health inequities, requires involvement from multiple sectors (i.e., medicine, public health, community and social services, education, justice, etc.). Because of this need for intersectoral collaboration, ‘joined-up’ (JUG) approaches to the governance of child health policy making may be required.

Governance refers to patterns of rule, including the arrangements, institutions and norms that influence how decisions are made [3]. Importantly, this is not solely about the actions of public authorities, but of how these authorities work together and with non-governmental actors to coordinate policy attention and activity. Governance systems vary in terms of who is involved, where or how stakeholders interface, and in the actions that result from their joint decision-making processes [4]. Integrated governance is defined as “an action initiated and developed by a public agency striving to integrate the actions of other actors around the same problems” [5]. Recommended for the coordination of policy making efforts in the face of challenges that cross government sectors, such JUG approaches (also called whole-of-government, holistic, or horizontal government) [6,7,8] are appealing as a solution to the challenge of child health policy making. Yet despite much lamenting about fragmented government being a problem, the literature examining solutions is sparse [9]. Scholarship from public administration has examined theories of institutional change, including the concept and value of JUG since the late 1990s [10], yet there is little in terms of applied literature examining implementation or effectiveness, particularly with relevance to child health.

Intersectoral action and associated forms of JUG may be especially relevant in efforts to optimize early child development. The limited professional intervention in children’s early years, between infancy when children regularly see medical or public health professionals, and school age, when educational professionals intervene, results in gaps in what is known about how children are faring. These gaps have led to repeated calls for better data and strengthened interventions. Proponents aim to advance intersectoral solutions, to address the complexity of early child development, and to account for the social determinants of health [11,12,13,14]. Developmental screening (use of standardized tools to identify children at increased risk for delay and disorder before school entry) has emerged as a prominent, if contested, strategy to address the gap in the early years. Though not supported by evidence review bodies as a form of population screening [15,16], professional societies such as the American Academy of Pediatrics and the Canadian Pediatric Society have strongly endorsed the practice [17,18]. Moreover, developmental screening has been taken up in various forms in a number of countries [19,20].

The case of developmental screening illustrates the complexity of child health policy making. It evokes multiple agendas for action, including the specific medical aim of early detection of delay or pathology as well as more expansive social policy aims related to parent–child bonding and the prioritization of children’s needs in public policy [21]. In addition, the case of developmental screening raises questions about how governance arrangements can navigate such diverse purposes, to assess and prioritize policy options for intervention. Part of what is at issue is how actors and their interests are channeled—in ways that enable or constrain the efforts of actors within or outside of government, who may seize an opportunity to influence policy outcomes. These actors are typically thought of as policy entrepreneurs, but as we will suggest, such agents might also be thought of as policy rogues by departing from authorized channels of decision making [22,23,24,25]. To explore these questions, we studied how two provinces in Canada approached child health governance and addressed the policy option of developmental screening. Focusing on Ontario and Manitoba, our objectives were to (i) describe approaches to the formulation and design of child health policy (i.e., governance arrangements); (ii) explore the relationship between governance arrangements and agenda setting in the developmental screening context; and (iii) compare and contrast findings across jurisdictions.

## 2. Materials and Methods

### 2.1. Study Context

Canada has no national structure for the governance of child health. Most health and social services are under provincial jurisdiction but share a broad health policy framework under the terms of the Canada Health Act. Manitoba (pop.1.3M) is unique in having a cross-departmental strategy for child health (Healthy Child Manitoba) and was purposely selected as a case for analysis because it provides a way to examine a formally JUG approach. At the time of data collection, Ontario (Canada’s largest province, pop. 14M) had a standalone ministry for children that controlled developmental services. Ontario was selected as a case for analysis because it was the only province to have introduced a province-wide developmental screening program. Called an “enhanced well-baby visit” (EWBV) and delivered at 18 months of age, it involves the use of a health supervision guide (i.e., Rourke Baby Record) and a developmental screening tool (i.e., Nipissing District Developmental Screen). In Manitoba, developmental screening was on the policy agenda, but no decision about whether or not to universally implement the intervention had been made at the time of our study.

### 2.2. Design and Setting

Using key informant interviews and document review, we conducted a comparative policy analysis, to compare the distinct approaches to child health governance generally and to developmental screening policy specifically. Ontario and Manitoba provide an opportunity for rich comparative analysis given that they both expressed a desire for JUG approaches to child health but have approached policy for developmental screening differently and display distinct arrangements for governing child health. Case study methodology is appropriate because it enables development of a comprehensive understanding of a phenomenon within a particular system or context.

### 2.3. Key Informants

Findings are based on an analysis of Canadian policy documents and interviews with forty experts in child health, representing Canadian leaders in medicine, research and policy making. Interview respondents were drawn from the Canadian child health policy community, and identified through national and provincial policy and professional networks, as well as from policy documents and key informant recommendations. Potential respondents were sampled purposively for: (a) individuals who could speak to the nature and sequence of events associated with provincial policy considerations for developmental screening; and (b) individuals who could provide their opinions and experiences regarding policy formulation, design, and delivery, as it related to early child development initiatives.

Following established qualitative research methods [26], sample size (n = 40) was based on what is estimated to achieve both breadth in terms of facts about jurisdictional contexts and policy processes, and depth or redundancy in terms of emergent themes (i.e., when new interviews cease to provide new ideas or information).

Respondents were ascertained between 2014 and 2015 from four professional categories: medicine, research, government, and advocacy (see Table 1). The four categories were not mutually exclusive, and several interview respondents fit in multiple categories. Efforts were made to include similar representation from each province in terms of the various roles represented. Additionally, the number of interviews was proportional to jurisdictional size (i.e., twice as many interviews in the much larger province of Ontario). Potential participants were invited to participate by email-based letter with up to two reminders and each informant provided written informed consent. The University of Toronto Research Ethics Board approved this research.

### 2.4. Data Collection and Analysis

Case studies use multiple sources of information and depend on theory to provide an analytical structure and help organize empirical observations. Data collection strategies included document analysis and key informant interviews with child health experts.

#### 2.4.1. Documents

We searched published and grey literature (i.e., governmental strategic and annual reports, professional guidelines and recommendations) published between 2000 and 2015. The search strategy involved keyword searching (i.e., child health, governance, evidence, development, screening, surveillance, assessment, public health, population etc.) of electronic databases (including: Canadian Research Index, Government of Canada Publications, Scholar’s portal, PubMed, Google Scholar), citation searching, and manual searching of professional association and stakeholder websites. Our focus was on documents that could provide insight into: (i) the formal child health policy making process in Ontario and Manitoba; (ii) governance arrangements within child health systems (i.e., accountabilities, organization of programs, and structures within government ministries, and departments and service delivery organizations); and (iii) child health agendas and priorities. The majority of policy documents were gathered before interviews but a few were collected as a result of speaking to key informants who shared or directed us to additional sources they thought would be informative (e.g., agendas, meeting minutes, and proceedings; group recommendations/statements; terms of reference, expert group membership lists).

Documents contributed in three specific ways: (a) providing critical background information that helped identify key informants, context for the interviews, and interview questions; (b) providing historical information and chronology of events related to policy decisions, outlining government priorities and key stakeholders, all of which contributed to the creation of case descriptions; and (c) enabling cross-case analysis by providing additional data to what was collected during the interviews as well as corroborating accounts from interviews.

#### 2.4.2. Key Informant Interviews

Interviews were conducted in the period 2014–2015 and averaged one hour in duration (40–75 min). Interviews were open-ended, and semi-structured. The aim was to understand experts’ experiences with and perspectives on the child health policy and practice environments within which they operated.

Questions were tailored to each respondent’s position and expertise, based on a common interview guide that was informed by central themes identified in the document analysis and policy studies literature (e.g., the policy cycle; the ideas, interests and institutions represented within a policy system; problem definition and agenda setting). Interviews explored questions related to: the governance, organization and delivery of child health policy, decision-making processes related to developmental screening, and approaches to evidence review for child health policy.

All interviews were recorded, transcribed verbatim, and organized using a qualitative data management and analysis software [27]. Transcripts were analyzed using thematic analysis, drawing on the principles of constructivist grounded theory, and applying an iterative, constant-comparative method [28,29]. Similarities and differences in respondents’ attitudes, values and practices were compared within and across transcripts. There was redundancy in data for presented themes, suggesting we had reached saturation [29]. To ensure confidentiality of informants within a relatively small policy network, quotes were attributed to the role specified by respondents.

#### 2.4.3. Data Integration and Analysis

Analysis was pursued at several levels. First, we looked at each type of data and each jurisdiction separately (i.e., documents for ON; documents for MB; interviews for ON; interviews for MB). Second, data from documents and interviews were integrated to construct a detailed narrative that described each jurisdiction’s approach to governing child health—a case description. This triangulation of data sources enabled us to identify both converging and diverging accounts of event chronology (i.e., milestones and evolution of policy choices and processes), and to note inconsistencies and variations within a theme or jurisdiction. Third, we conducted cross-case analysis, drawing on previously identified descriptive categories and themes and noting where findings aligned with ideas and theories from the policy studies literature, particularly scholarship that addresses agenda setting, the use of evidence in health decision making and processes and structures for intersectoral approaches to governance [30,31,32,33,34,35,36,37].

## 3. Results

We first review the two provinces’ governance arrangements for child health, and second, how they responded to the specific policy issue of developmental screening. Findings are based on document analysis (n = 54; see Table 2 for details) and interviews with 40 key informants (Ontario: n = 27; Manitoba: n = 13; see Table 1 for details).

### 3.1. Governance of Child Health: Ontario and Manitoba

Both jurisdictions perceived the intersectoral nature of child health as a challenge. Noting that children span ministerial boundaries, respondents consistently argued that intersectoral and collaborative approaches to child health policy were needed. As respondents noted, “You can’t do [child health] within one ministry. It has to be an inter-ministerial approach” (ON19: Physician and Scientist). Yet it was challenging to organize policy attention in coordinated ways:

*We’ve deconstructed the child and given the health of the children off to the medical profession, and we’ve given the learning of children off to the education profession, and we’ve given behavior, social functioning to social scientists and psychologists, and sociologists. And everyone’s done their own work, and very interesting work, good work, but the child doesn’t develop within all these silos; it develops holistically*.

(ON10: Scientist)

Policy approaches could yield improved outcomes where “children aren’t cut up in different ministries.” (MB13: Scientist). Though it was challenging to craft a solution, government ministries and child health professionals and advocates had “to figure out a way to work seamlessly on their [children’s] behalf” (MB6: Senior Bureaucrat, HCM). Sharing this challenge and sharing the intent to join up policy approaches, both Manitoba and Ontario worked to establish governance models for child health that addressed the need for intersectoral collaboration. Yet they took different approaches to the organization of decision making and the representation of interests.

In 2000, the Government of Manitoba established the Healthy Child Manitoba (HCM) strategy, a cross-sectoral vision, delivered through a legislated Cabinet Committee [38]. Rather than creating a new ministry, the HCM strategy created cross-departmental capacity, through the Healthy Child Committee of Cabinet (HCCC) comprising 10 Ministers, representing Health, Education, Children and Youth, Family Services, Aboriginal and Northern Affairs, Housing, Labour and Immigration, Seniors, Jobs and the Economy, and Justice. HCCC was designed to facilitate “interdepartmental cooperation and coordination with respect to programs and services for Manitoba’s children and families” [39]. It was supported by the Healthy Child Manitoba Office, which coordinates program and policy development and the implementation and evaluation of Manitoba’s child-centric public policy within and across departments [39].

In 2003, the Government of Ontario created the first new ministry in 20 years—the Ministry of Children and Youth Services (MCYS, now Ministry of Children, Community and Social Services), with an independent organizational structure and budget. MCYS’s stated purpose focused on service integration: on bringing programs for children and youth “under one roof” to better serve children and their families [40]. The creation of the new ministry involved the transfer of legislated responsibility for child-centric programs and services from the Ministries of Community and Social Services, Health and Long-Term Care, and Community Safety and Correctional Services to MCYS. Additionally, explicit in the mission of MCYS was a commitment to “work with government and community partners” to “give children the best possible start in life” [41].

Based on the vision articulated in ministry websites and strategic plans, MCYS and HCM shared a broadly similar vision (Table 3): emphasizing the need for children to realize their full potential, fostering collaboration with other government and community partners, and focusing on principles of inclusion, collaboration and accountability. Despite the similarities in vision, the scope of activities differed across the two jurisdictions. MCYS oversaw the administration of multiple statutes and had fiduciary responsibility for multiple programs for children 0–18 years. HCM had fiduciary responsibility for grants and, like MCYS, coordinated policy and programs across early childhood and adolescence. By contrast, HCM prioritized the prenatal–preschool period and also emphasized evidence-based policy making. The HCM Act mandated that government monitor and report regularly on the effects of the HCM Strategy, measuring whether HCM policies and programs were working to improve defined outcomes [38,39]. The Manitoba Centre for Health Policy played an essential role in generating and reviewing evidence to support HCM’s evidence-based policy making mandate.

### 3.2. Coordination Across Sectors for Child Health

#### 3.2.1. Manitoba: Aligning Interests

Respondents in Manitoba were united in their support of a JUG approach to child health, emphasizing the value of a system where coordination is formalized. The creation of HCM, where the alignment of interests is encoded in legislation, was a point of pride, particularly among policy makers. Respondents described a collaborative culture, where each ministerial voice was valued equally in support of “the common good for kids” (MB7: Senior Bureaucrat).

The Manitoba Centre for Health Policy (MCHP), where population data are housed and analyzed to inform policy evaluation and agenda setting, was felt to be key to successful governance. The MCHP was understood as a strong partner to HCM, with datasets on welfare, education, justice and “all the sectors that touch on children” in the Population Health Research Data Repository. Partially attributable to the Healthy Child Committee of Cabinet, MCHP had been collecting administrative data across sectors for decades and was thus able to conduct independent research that was used to answer complex questions for bureaucrats. This partnership was the foundation for evidence-based policy making.

The opportunities afforded through shared budgets and shared data were mentioned repeatedly. Comparing themselves to other governments across the country, one policy maker explained how Manitoba differs: “The question we often pose is: if you had the choice at a cabinet table of having 10 voices and Ministers speaking on behalf of something or a super Minister of one, what would you choose? And that’s not a difficult answer, right?” (MB6: Senior Bureaucrat).

Importantly, collaboration was seen to be central to HCM’s operations and decision-making processes. Respondents working within that environment were reflective about how intersectoral collaboration requires immense effort, because fundamentally, intersectoral governance is about “strengthening relationships” and “getting existing barriers out of the way” (MB7: Senior Bureaucrat). As one senior leader shared: “It is more work and it’s easy for people to go rogue and do things on their own and we will be the first to admit that that hasn’t entirely ceased despite the intent of the legislation enabling people to work together—it’s not easy”. They continued to explain that the positive outcomes justified the effort to address the challenge: “But there’s absolutely no comparison in terms of the positive outcomes we can have for kids in this system compared to the other [days before HCM]” (MB7: Senior Bureaucrat).

Another senior bureaucrat echoed the conviction that the intersectoral policy making model is effective, proclaiming “it’s the only way to do business” (MB11: Senior Bureaucrat). Additionally, there was an expressed pride that the collaborative approach simply works: “I think there’s a lot more cross-pollination in Manitoba, and when I talk with people from other provinces I realize how lucky we are”. The effectiveness of this approach was attributed to a culture where people know and respect one another and where it is commonplace to: “just pick up the phone or shoot an email to some of these high-level policy makers and actually get responses” (MB1: Scientist).

Echoing that interaction between bureaucrats and scientists was normalized, researchers described their interactions with Ministers as a forum for “honest, real and direct conversations” that were solution-focused. There were regular opportunities where “ministers can talk to each other and hopefully develop policies that are intersectoral and reminds them that there’s not just their department and their budget” (MB13: Scientist). Manitoba’s approach involved evidence appraisal and joint problem solving. The focus on shared decision making and the philosophy of collaboration were seen as means to more efficacious investments for children.

#### 3.2.2. Ontario: Disjointed Interests

In stark contrast to the experience in Manitoba, Ontario respondents described a policy environment where interests were not aligned and where each ministry connected to children remained quite separate from the other. As a result, the reality of governing child health in Ontario was perceived as chaotic at times, with respondents highlighting the seemingly arbitrary divisions of responsibility. For example, thinking about childhood disability, one expert said: “There’s an artificial divide between ‘oh, we only do assessment in the Ministry of Health and we only do intervention through the Ministry of Children and Youth Services’, so everybody just keeps handing the ball back and forth” (ON19: Physician; Scientist).

The multitude of players and lack of clear structure to coordinate their interactions was felt to be an overarching barrier. Another key concern among respondents was a pervasive sense of competition between sectors, that many characterized as “turf protection”. A number of respondents voiced surprise at the lack of collaboration and coordination for the common good of kids, for example: “A lot of people are invested in their own world and in their own cause or their own group, in their own sector” (ON14: Senior Bureaucrat). Similarly, the size of Ontario’s bureaucracy was portrayed as problematic, with few mechanisms to communicate across sectors, resulting in duplicative initiatives. For example, one respondent described the impossibility of obtaining a comprehensive understanding of government programs to address childhood obesity. Having asked a seemingly “simple question” about current programs across government, civil servants:

… *said we might be able to do it for the Ministry of Health, but even that might be difficult. But I’m not sure we can do it across government. Now in the end they did come with this, pages and pages and pages, and I don’t know how they got it, or where they got it from, but it was… dozens and dozens and dozens, but none of them were linked. And I bet virtually none of the people doing these different programs knew about the other programs, and it was like you get a shotgun and just shoot it against the wall, and splatter. And I’m not blaming them…it’s actually really hard to integrate. I think governments understand this and they genuinely want to do the best thing… they want to help children in the best way, and they understand that they’re siloed, and they understand that they need to overcome those silos. But like I said it’s hard. They probably don’t know how to start going about it other than to have a couple of meetings to talk about it, and they’re busy just delivering programs*

(ON10: Scientist)

In spite of some expressed sympathy for the challenges inherent to the operation of a very large bureaucracy, a critique related to communication failures persisted. Here, respondents were again concerned with competition in a context where one Ministry “wins” ownership of a particular cross-sectoral child health project (e.g., mental health): “Our health care system, our governance of child health and well-being fits into the Ministry of Education, Ministry of Health, Ministry of Child and Youth Services, Ministry of Social Services…and they don’t come together to talk the same language, they all vie for funds for their pet projects” (ON12: Pediatrician; Scientist).

Notwithstanding the clear sense of fragmentation and disjointed interests in Ontario, a few vocal respondents expressed skepticism at structural solutions such as “joined-up nonsense” (ON25: Senior Bureaucrat), claiming instead that changing political culture is a far bigger barrier to effectively coordinating governance for complex problems. Noting that culture is very difficult to change, one respondent declared: “Why do you think they change structure all the time? Because it’s easy. Doesn’t make any difference, but it’s easy, and it gives the impression that it’s changing something” (ON25: Senior Bureaucrat). This respondent remarked that the variety of governance approaches nationally as well as the multitude of ways Ministries are “lopped and split” by topic or population are proof that solutions to intersectoral challenges remain elusive. If there were an ideal system, these respondents suggested, every jurisdiction would replicate that approach.

### 3.3. Approach to the Case of Developmental Screening

One of the important things that both HCM and MCYS oversaw was a set of screening interventions in early childhood. Both jurisdictions had parallel screening systems in infancy with universal newborn bloodspot screening administered through the health system. At the time of data collection, Ontario also had universal hearing screening, whereas hearing screening was in development and not yet universal in Manitoba. As well, both jurisdictions operated similar prevention and early intervention home visiting programs for new mothers, administered by Public Health nurses. A third time point occurred in both provinces to assess school readiness and was administered through the education system in Kindergarten [42]. This mix of screening interventions nonetheless was seen to leave a gap between infancy (ending at 1 year of age) and kindergarten (beginning at age 4), providing parallel contexts for considering the policy option of developmental screening.

In 2011, the Canadian Pediatric Society issued two related policy statements, which together called for greater investment in early child development. One focused on longitudinal monitoring and measurement, arguing that “early childhood counts” and therefore needs to be counted [43]. The other endorsed “enhanced developmental assessment at 18-months” involving a shift in practice from a traditional well-baby check-up to a systematic and “pivotal assessment of developmental health” using standardized tools that can “facilitate a broader discussion between primary care providers and parents” [18]. Both sets of guidance aimed to fill a piece of the “gap” in early childhood. Study respondents echoed the need to correct this “missed opportunity,” noting that, from a population health perspective, the EWBV is “a great opportunity for surveillance in terms of getting that information” (ON24: Senior Public Health Administrator). Despite a shared view of an assessment gap, the two provinces responded quite differently to the policy option of developmental screening.

#### 3.3.1. Manitoba: Focus on Structured Process

In contrast to Ontario’s policy action of implementing the EWBV in 2009, six years later, developmental screening was still under policy consideration in Manitoba. In 2015, HCM published a 5-year action plan for ECD that recommended establishing a cross-departmental committee to explore the feasibility of implementing a provincial developmental screening program [44]. At the time of data collection, the committee had been convened and had sought representatives from a number of sectors, including Aboriginal, public health, childcare, developmental services, research, early education, fetal alcohol spectrum disorder, and population health equity groups. Interview respondents anticipated that some form of developmental screening program at 18 months would be implemented and suggested that delivery via public health would be more likely than through primary care, as in Ontario: “Something that doesn’t get a lot of attention is that it doesn’t have to be doctors who do it” (MB2: Researcher). However, the specifics of any program or timeline remained undetermined and contingent on a thorough review of the evidence and consideration of alternative policy options.

Manitoba respondents tell a story of due process, and interest alignment—fostering collaboration between the practitioners delivering services on the ground, the scientists who were generating evidence, and the bureaucrats from across relevant ministries. Though they had not yet decided on whether or how to implement developmental screening, all respondents voiced an intention to apply their regular process of collaborative deliberation, evidence adjudication and policy consideration to the case. Most striking in the Manitoba context was a focus on stakeholder engagement. Deliberation of evidence and consensus generation were understood as routine and implicit in the policy and service design process. For example, in considering how the policy design for developmental screening would transpire, respondents described the strategy as replicating their usual process, noting the imperative of broad engagement and a wide consideration of options for implementation: “[We are] looking at a wide range of gaps and needs and complications and concerns [in ECD] and potential synergies so we really want as many folks around the table as possible to help us answer those questions” (MB10: Senior Bureaucrat). Similarly, in contemplating the potential design and delivery of a screening program respondents referred to the routine nature of engaging with varied stakeholders and consulting with data as established norms in the policy process: “we would probably follow the same kind of unfolding and evolution we have in other places in terms of how that works with partners, and how the data would be used” (MB7: Senior Bureaucrat, HCM).

Despite the routine nature of engagement and evidence review, the political environment was acknowledged to mediate policy uptake. Leaders within HCM noted that the fiscal environment following the financial crisis in 2008, and particularly the previous few years (2012–2015), was vastly different compared to the previous decade and seen as potentially impeding the ability to embark on new programs such as developmental screening. However, the expectation that implementation may not be economically feasible in the short-term did not forestall planning and consideration of ultimate program design.

As previously noted, the role of provincial population and program evaluation data was central to Manitoba’s decision-making process. Respondents emphasized the links between science and policy again and again, referring to HCM being “at the intersection of policy and the whole research and evaluation world…part of our job is to be that bridge between government and community” (MB6: Senior Bureaucrat). Ongoing program evaluation was seen to be inherent to agenda setting and resource allocation in terms of both investment and disinvestment decisions. Decisions about policy change were contingent on evidence of value—and that evidence often came from linked provincial datasets. As one research scientist explained, the Manitoba Centre for Health Policy relied on data to “either strengthen or back away from a program that is not being effective” reiterating the importance of considered decision making.

In predicting how developmental screening might be implemented, respondents emphasized that the core values of partnership and evidence-based policy making would similarly be core to policy deliberation, stating “policy follows purpose” (MB7: Senior Bureaucrat). In sum, implementation was grappled with throughout the policy process. Indeed, decision makers could not move forward without consideration of it, nor without consultation with relevant partners. While respondents acknowledged this process to be time-intensive, the result of broad stakeholder buy-in and robust data generated confidence in the system and pride in the rigour of their policy process.

#### 3.3.2. Ontario: Focus on Action

In 2005, MCYS created an expert panel comprised of 21 senior leaders in child health to advise on implementation options for developmental screening. The expert panel convened medical practitioners, child health service provider organizations and government executives over a period of eight months to review and develop potential strategies “to involve primary care providers in monitoring and promoting healthy child development”, aiming to “create a culture that enhances the developmental health and well-being of children” [45]. The panel’s report recommended implementation of the Enhanced Well-Baby Visit at 18 months across Ontario within two years, and outlined six strategies for implementation: (1) provide tools (specifically, the Rourke Baby Record and Nippissing District Developmental Screen); (2) build effective partnerships between providers and parents and community resources; (3) provide education and support for primary care providers; (4) measure and manage wait times for child development services; (5) describe the developmental health status of children; and (6) evaluate the impact of the 18-mos visit [39]. Following the expert panel report, the Ontario College of Family Practice (OCFP) with funding from MCYS, created an 18-month Steering Committee, composed of eight expert clinicians. This Steering Committee was supported by the OCFP Guidelines Advisory Committee to make evidence-based clinical practice recommendations in support of the expert panel report recommendations. The Steering committee “sought to find evidence of practical benefit to the time-stressed clinician: specifically, feasible office-based interventions that might have a significant impact on the detection or improvement of child development” [46]. Anticipating that changing physician practice patterns may be challenging, the OCFP also developed a practice guideline document and funded two EWBV pilot projects to test implementation strategies [47]. Almost ten years later, at the time of the interviews, Ontario respondents felt that recommendations of the expert panel—to provide the tools and the education for providers (1) and (3)—had been implemented to a greater degree than the partnerships (2) and that the measurement and evaluation pieces for the health system, child development and program impacts (4–6) remained in development.

**Figure 1:**
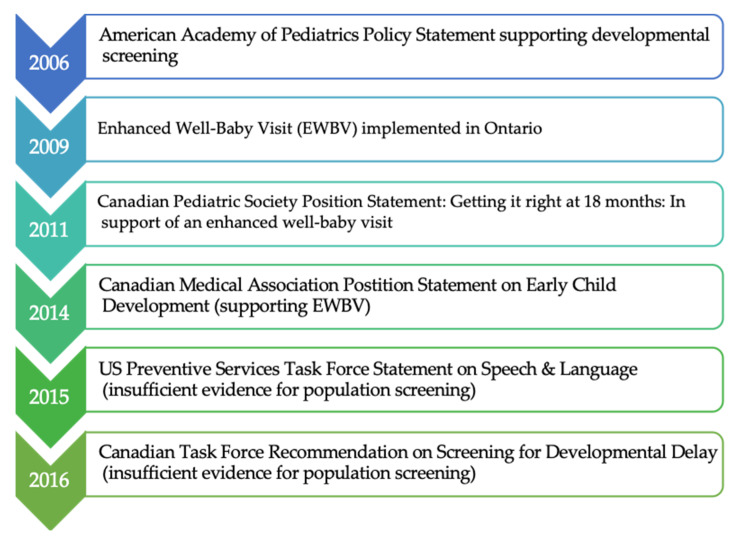
Timeline of Guidelines and Position Statements Related to Developmental Screening and Policy Uptake of the EWBV.

In 2009, unique in Canada at the time, Ontario began delivering the enhanced 18-month well-baby visit as a universal service, through primary care [18,48,49]. Program design and delivery was led by MCYS. In consultation with the Ministry of Health (MOH), a fee code for physicians was established, which doubled the previous payment. This was in advance of the 2011 Canadian Pediatric Society guideline recommending an EWBV at 18 months, and ahead of guidance from evidence review bodies (Canadian Task Force for Preventive Health Care) which came in 2016 (Figure 1).

In discussing the case of developmental screening, respondents were as critical of the non-collaborative nature of Ontario’s policy process as they had been for the overall approach to child health governance. Central to the narrative of turf and competition between the MCYS and the MOH was the fact that MOH had a much larger purse and far more expertise than MCYS—a relatively new entity—in terms of policy formulation, physician engagement and the implementation of screening programs. According to some respondents, MCYS could have drawn on MOH’s experience in designing and implementing the policy but chose not to: “This was the first go-around for MCYS and I think that it was to MCYS’s detriment that they didn’t include the MOH people, but they really wanted to be seen as the ones who owned it” (ON2: Physician; Policy Advisor).

Though MCYS may have aimed to own the program, the decision to implement the enhanced well-baby visit did not come from MCYS alone, but rather was largely credited to action by a few, individual child health advocates. These few key individuals also contributed to the guidance documents and policy statements supporting early intervention. These champions were said to have “gotten docs on board” and garnered political support for the aim of intervening earlier, suggesting that the 18-month visit was “an opportunity” to seize. Describing how Ontario achieved policy change, one respondent explained:

*I think it’s one: about the timing. And I think a very, very big part of it is about the champions. We would not have the 18-month Well-Baby Visit without [them]; the enhanced 18-month, we just wouldn’t. As well within the Ministry we have people who really got it and have been a champion for it right all along; their energy, passion, had a big part in why it unfolded*.

(ON22: Physician; Policy advisor)

Informants were candid about the strategy employed for the creation of the Ontario EWBV expert panel, noting that efforts were made to ensure representation from the provincial medical association, to establish support for the fee code, the provincial College of Family Physicians, to garner professional support, leaders in Pediatrics, who could champion the value of the visit among their profession, and importantly, a key senior leader from within MCYS. As one central member admitted: “it was strategically set up, it wasn’t totally random and there were some physician leaders, pediatrician leaders that, we knew that if we had them on board, we knew it would be way easier” (ON12: Pediatrician; Senior Bureaucrat).

Respondents suggested that individual champions could tip the balance in support of the EWBV because the other system conditions were ripe in terms of resources. As one respondent noted, “it was a small potatoes thing” (ON3: Pediatrician; Researcher) compared to other investments the Ministry of Health was making and it “was in the days when there was money” (ON2: Physician; Policy Advisor). Not only was the fiscal environment not a significant hurdle at the time, the cultural environment was felt to be especially conducive to interventions aimed at optimizing the early years, in part because Ontario had recently implemented full-day kindergarten, paving the way for population-level investments of this sort.

This set of financial and cultural conditions were seen to provide a prime environment for action. This was paired with the perceived problem that too many children were arriving at school with vulnerabilities [18], and the opportunity that primary care offered as a venue to act on behalf of a large portion of children: “here we had an opportunity; here’s a platform where all the kids are seen” (ON22: Physician; Policy advisor). The notion of seizing the opportunity to act was characterized as “not asking for much”, with another respondent speculating that it “should have been an easy sell” to decision makers.

Reflecting on the years since then, respondents also noted the labile nature of the political climate—that the constrained public purse post-2008 meant that investments in preventive child health were unlikely to gain traction at the time of the interviews. Echoing what had been voiced in Manitoba, a respondent noted that in the current (2014–2015) fiscal climate, there would likely not be the same ability to generate policy uptake for developmental screening.

The two jurisdictions also had very different approaches when it came to review and use of evidence to inform developmental screening policy. In contrast to Manitoba, where evidence appraisal was formalized, sources of evidence in Ontario were more varied and were drawn on opportunistically. For example, many respondents noted that there were multiple justifications for intervening early, including the growing science of brain development and the promising findings from international healthy start programs, focused on preschool education and social welfare policies [50,51,52]. In the Ontario context, these sources of evidence were seen to provide pressure for decision makers, compelling them to act: “I think that was really the impetus that pushed the government in the direction of saying, we have to do something about healthy child development, promoting healthy child development, and it’s good for Canada if we do” (ON19: Physician and Scientist).

In contrast to Manitoba, evaluation capacity was not described as an important input to policy making in Ontario. Despite momentum to implement, garnered by key champions and opportunistic timing, the limited ability to collect data and evaluate implementation was a dominant criticism of the Ontario experience. First, from the perspective of program oversight, many remarked on the lack of accountability mechanisms, particularly in terms of tracking and ensuring that physicians were conducting a thorough visit, and billing appropriately. As one senior person very close to the roll-out lamented: “We have no fricking clue what is actually happening on the ground” (ON22: Physician; Policy advisor). Beyond tracking how many physicians had received continuing education on the tools or billed using the fee code, there was no means to track how often the assessment tools were completed. This was seen as an accountability gap, with frustration that physician compensation had not been tied to data collection: “You should have had to do more. Right now, you do the visits and you bill for it; it should have been: do the visits, document the visits, submit that document, and then you get the money” (ON9: Scientist).

The role of evidence also differed by jurisdiction when looked at from a program evaluation perspective. Signaling the challenge in terms of policy learning, in Ontario, data collection and program evaluation was seen to be an after-thought, with the systematic means to measure quality (e.g., referral pathways, impacts on waitlists, child health outcomes) as secondary to policy action: “In terms of research design, we were pulling out our hair because this got rolled out, as a government initiative, not as what would be the most effective way to change physician and parent behaviour” (ON22: Physician; Policy advisor). Additionally, as voiced by someone within the Ministry of Children and Youth Services:

*It was a very strange thing that we ended up rolling it out and I was [in a senior role] at the time so I can’t really criticize everybody on the fall-out of getting it all implemented…and then I started to say, OK and so how do I get the data back and it was sort of like, oh—oops we forgot to figure this part out*

(ON17: Senior Bureaucrat)

## 4. Discussion

The complexity of policy making for preventive child health is understood as a wicked problem because it straddles the boundaries of health and social policy. In an effort to overcome the failures, redundancies and inefficiencies that can result from the traditionally siloed nature of social welfare systems that support health and wellness through diverse departments (e.g., health, education, social services), intersectoral approaches to governance are appealing. Yet JUG is a challenge in intersectoral contexts such as child health because there are so many actors and interests involved. Integrated governance requires joining up of structures and institutions, including integrating how actors and their interests are managed, and how ideas, and the agendas they motivate, are coordinated. We conducted a comparative policy study to explore whether and how JUG was mobilized across two provinces in Canada as a strategy to support healthy child development and resolve contentious policy options such as developmental screening. Our findings reveal two distinct stories of governance arrangements to meet the needs of child health. While both provinces intended to be joined-up in principle, only one was joined-up in practice.

In Manitoba, the process and structure for child health policy making was formalized and clear, collaboration between ministries was embedded in the agenda-setting process, and data were used to inform decision making as well as ongoing program evaluation. In short, Manitoba appears to have achieved a JUG system in practice, with concerted efforts to overcome silos and turf protection and block the kind of independent agenda setting action that is generally seen as policy entrepreneurship, but which was seen by Manitoba informants as “going rogue”. The idea of the ‘policy rogue’ emerged from the data and is not an established counterpoint to the idea of the policy entrepreneur, though there is some literature suggesting a move in that direction [53]. The concept is useful in highlighting the important ways in which the image of a policy issue—whether positively viewed as entrepreneurial effort, or more negatively seen as rogue action contravening authorized processes—very much depends on the perspective from which this is viewed [54].

Manitoba’s formalized decision process considered implementation and evaluation up front, and importantly, approval was conditional on an intervention being judged able to work. In contrast, in Ontario, despite a remarkably similar intention to create a JUG system to support child health, there was a non-system in practice—policy processes were opaque, relationships were fractured, the culture was competitive and turf protection was abundant, with limited capacity to measure the impact, effects or quality of policy decisions that had intersectoral reach. In contrast to Manitoba, these contexts provided opportunity for independent agenda setting action—efforts that were positively viewed, in the vein of policy entrepreneurship (not policy rogues).

In terms of how governance arrangements were organized to attend to the specific case of developmental screening, Manitoba applied an established process of broad cross-departmental engagement, with an emphasis on aligning interests and a clear process for consideration of authorized evidence. At the time of data collection, this process was underway and no policy decision about developmental screening had been made. In Ontario, by contrast, policy action was directly tied to the absence of JUG in multiple ministries that serve children. The absence of a formal means of interacting left a system that was disjointed, permitting policy entrepreneurs to implement change. Since it was opportunistic it may be unsurprising that key details, such as mechanisms for program evaluation, were not worked through in advance. 

There are potential benefits and challenges to each approach. For Ontario, given the environment of limited evaluation capacity, demonstration of the value of developmental screening may prove challenging, undermining the larger aspiration to invest in kids. However, the experience of Ontario in not having achieved integration is by no means an outlier. Indeed, much of the literature supports that ‘doing’ JUG is difficult and scholars have demonstrated that politics can easily undermine structural solutions [55,56] with similar challenges seen in other areas of child health policy making [57].

Conversely in Manitoba, evidence adjudication was baked into the process for policy deliberation; evidence-informed policy making was a central focus and the partnership with the Manitoba Centre for Health Policy and HCM was core to HCM’s legislated operation. This approach of considered decision making, with a reflective data-to-policy evidence cycle means that decisions were based on a high degree of consultation and evidence review throughout all phases of policy development and implementation. The implication of this process was inaction during a policy window that may have passed. This may be perceived as positive in terms of not wasting resources on something unproven, or negative in terms of missing an opportunity to support child health in one way or another.

Through the lens of evidence-informed policy making, the implementation of JUG in Manitoba supported a collaborative and evidence-informed process. Yet the relationship with the Manitoba Centre for Health Policy authorized certain types of evidence (e.g., medical literature; administrative data), whereas the lack of a formalized process in Ontario made different kinds of evidence inputs possible [58,59,60]. This is relevant because, as others have argued, governments use evidence selectively and scientific evidence alone is insufficient to drive policy change; problem framing is also critical [61,62,63,64].

We are left asking: how did children fare as a result of these distinct choices? While the benefits to children are a crucial way to evaluate policy, the outcomes remain unclear and open to multiple interpretations. For developmental screening specifically, it depends on the agenda that the intervention aims to serve (i.e., for those subscribing to a classic screening agenda of identifying abnormality, outcome metrics require evidence of clinical effectiveness, including high sensitivity and specificity of the tool) [21]. We must note that our findings only account for a brief timeframe in an ongoing process, and there are many interacting factors in a complex system.

We acknowledge several other limitations. A central limitation of this work is uncertainty about whether and how jurisdictional size makes one model of governance more or less feasible. One could argue that, based on size or history, Manitoba was fertile ground for the creation of a JUG system, thus we may be drawing unfair comparisons. Additionally, we acknowledge that JUG in Manitoba was somewhat theoretical as it applied to the case of developmental screening, and we cannot be certain about how things would evolve. However, we feel confident about our extrapolation to the case, because respondents were clear in portraying an established culture of collaboration and emphasizing how the requirement of integrated decision making applied to all child health policy making.

Reflecting back on the original governance vision, Ontario’s MCYS sought to bring together programs for the most vulnerable children. Since MCYS lacked power compared to the much larger Ministries of Health and Education, it is difficult to measure the significance of MCYS’s original aim to organize attention for vulnerable kids. Since 2018, a new government in Ontario has re-organized the Ministries that attend to children, eliminating MCYS, and creating a new Ministry of Children, Community and Social Services. In the new structure, developmental services and mental health are both carved off and allocated to different ministries, further fracturing decision making for child health and development, re-shuffling the needs of children around once again. The effect is that the governance of child health in Ontario is still not joined up, and the absence of structure now exists in another form. In Manitoba, HCM remains, despite a change in government. Thus, if stability is a measure of success, then Manitoba has performed well.

In sum, we find that although both jurisdictions had similar intentions of achieving intersectoral collaboration in pursuit of improved child health, the result of their actions was not similar at all. One created a standalone ministry and the other a cross-ministerial strategy. In so doing, the Manitoba system created a way to mediate across sectors and interest groups and the Ontario system did not. In effect, the Manitoba system curtailed rogue actors while the Ontario system enabled policy entrepreneurs.

## Figures and Tables

**Table 1:** Respondent characteristics.

Roles *	Ontario	Manitoba
Medicine ^1^	12	3
Research ^2^	13	6
Government ^3^	9	7
Advocacy/Other Service delivery ^4^	5	1
Total *	27	13

* Some respondents occupy or self-identity as belonging to multiple roles, and therefore the number of roles sum to more than the total number of respondents. ^1^ Specialist or general pediatricians, and leaders in child health. ^2^ Physicians or PhDs with an academic appointment at a university or research institute, pursuing research with critical relevance to child development or developmental screening. ^3^ Individuals at the frontline of formal policy decision making (i.e., Directors, Assistant Deputy Ministers, Deputy Ministers) within Ministries of Health or Ministries of Children. ^4^ Individuals who worked in organizations with a service and/or advocacy role or in senior positions within relevant professional organizations (i.e., Ontario Medical Association, Canadian Pediatric Society, Ontario Public Health Association).

**Table 2:** Documents analyzed.

Type of Document	Number
(a) National and provincial reports and professional position statements related to developmental screening and early child development (e.g., Canadian Pediatric Society, Canadian Task Force on Preventive Health Care, Canadian Medical Association, Ontario Medical Association)	14
(b) Ministry (MCYS and HCM) organizational documents (e.g., legislative documents, annual reports, strategic plans, expert panel reports, etc., that describe child health development priorities and programs within Manitoba and Ontario)	34
(c) Other relevant documents (e.g., conference presentations, toolkits, working papers) related to developmental screening in Ontario and Manitoba	6

**Table 3:** Child health governance structures in Ontario and Manitoba (2014–2015).

Jurisdiction	Ontario	Manitoba
Structure	Free-Standing Ministry	Cross-Departmental Strategy
Origin	Ministry of Children and Youth Services (MCYS) was created in 2003 to “make it easier for families to find the services to give kids the best start in life; make it easier for families to access the services they need at all stages of a child’s development; and help youth become productive adults.”	Healthy Child Manitoba (HCM) Strategy—a network of programs and supports, initiated in 2000. Set in legislation via HCM Act in 2007.
Vision	“An Ontario where all children and youth have the best opportunity to succeed and reach their full potential.”	“The best possible outcomes for all of Manitoba’s children.”
Mission and Goals	“The Ministry is working with government and community partners to develop and implement policies, programs and a service system that helps give children the best possible start in life, prepare youth to become productive adults and make it easier for families to access the services they need at all stages of a child’s development.”	HCM works across departments and sectors to facilitate a community development approach for the well-being of Manitoba’s children, families and communities. The priority focus is on the prenatal period through the preschool years. “To their fullest potential, Manitoba’s children will be: physically and emotionally healthy; safe and secure; successful at learning; socially engaged and responsible.”
Guiding Principles	Child and youth centred; responsive; inclusive; collaborative; outcomes-driven; accountable.	Community based; inclusive; comprehensive; integrated; accessible; quality assurance; public accountability.

## Data Availability

The data are not publicly available due to confidentiality concerns.
